# Understanding Potentially Preventable Mortality Following Oesophago-Gastric Cancer Surgery: Analysis of a National Audit of Surgical Mortality

**DOI:** 10.1245/s10434-023-13571-8

**Published:** 2023-05-08

**Authors:** David S. Liu, Aly Fayed, Penelope Evans, Tim Bright, Ahmad Aly, Cuong Duong, John Spillane, Laurence Weinberg, David I. Watson

**Affiliations:** 1 Upper Gastrointestinal Surgery Unit, Division of Surgery, Anaesthesia and Procedural Medicine, Austin Hospital, Heidelberg, VIC Australia; 2 Division of Cancer Surgery, Peter MacCallum Cancer Centre, Melbourne, VIC Australia; 3 General and Gastrointestinal Surgery Research and Trials Group, The University of Melbourne, Department of Surgery, Austin Health, Heidelberg, VIC Australia; 4 Flinders Medical Centre, Oesophago-gastric Surgery Unit, Bedford Park, SA Australia; 5 Discipline of Surgery, College of Medicine and Public Health, Flinders University, Bedford Park, SA Australia; 6 Department of Anaesthesia, Austin Health, Heidelberg, VIC Australia

**Keywords:** Mortality, Oesophageal, Gastric, Cancer, Surgery, Audit

## Abstract

**Introduction:**

At a national level, understanding preventable mortality after oesophago-gastric cancer surgery can direct quality-improvement efforts. Accordingly, utilizing the Australian and New Zealand Audit of Surgical Mortality (ANZASM), we aimed to: (1) determine the causes of death following oesophago-gastric cancer resections in Australia, (2) quantify the proportion of potentially preventable deaths, and (3) identify clinical management issues contributing to preventable mortality.

**Methods:**

All in-hospital mortalities following oesophago-gastric cancer surgery from 1 January 2010 to 31 December 2020 were analysed using ANZASM data. Potentially preventable and non-preventable cases were compared. Thematic analysis with a data-driven approach was used to classify clinical management issues.

**Results:**

Overall, 636 complications and 123 clinical management issues were identified in 105 mortalities. The most common causes of death were cardio-respiratory in aetiology. Forty-nine (46.7%) deaths were potentially preventable. These cases were characterized by higher rates of sepsis (59.2% vs 33.9%, *p* = 0.011), multiorgan dysfunction syndrome (40.8% vs 25.0%, *p *= 0.042), re-operation (63.3% vs 41.1%, *p *= 0.031) and other complications compared with non-preventable mortality. Potentially preventable mortalities also had more clinical management issues per patient [median (IQR): 2 (1–3) vs 0 (0–1), *p *< 0.001), which adversely impacted preoperative (30.6% vs 7.1%, *p *= 0.002), intraoperative (18.4% vs 5.4%, *p *= 0.037) and postoperative (51.0% vs 17.9%, *p *< 0.001) care. Thematic analysis highlighted recurrent areas of deficiency with preoperative, intraoperative and postoperative patient management.

**Conclusions:**

Almost 50% of deaths following oesophago-gastric cancer resections were potentially preventable. These were characterized by higher complication rates and clinical management issues. We highlight recurrent themes in patient management to improve future quality of care.

**Supplementary Information:**

The online version contains supplementary material available at 10.1245/s10434-023-13571-8.

Despite advances in surgical and perioperative care, low-volume high-risk procedures such as gastrectomy and oesophagectomy remain associated with significant postoperative mortality.^1^ Recently, the Australian Institute of Health and Welfare demonstrated that the Australian national postoperative mortality rate for oesophagectomy is 3.5%.^2^ Additionally, registry data from the state of New South Wales have reported a mortality rate of 4.1% following oesophago-gastric cancer resections.^3^ These rates are higher than major international centres, which typically report a mortality rate of under 2.5%.^4–6^

Whilst centralization of cancer services in high-volume centres may partly explain these differences,^7^^,^^8^ it is likely that many other factors contribute to variability in patient outcomes.^9^ To better direct quality-improvement efforts, it is important to understand the underlying factors that contribute to an apparently higher postoperative mortality rate in Australia.^10^ This is particularly pertinent in the Australian context, as centralization of cancer services has proven logistically challenging due to societal attitudes, a mixed public/private system, different levels of healthcare responsibility between State and National governments, and the vast geographical distances between townships in this country, with many individuals located remotely and some even > 3000 km from the nearest large city.^11^

Although cancer registries and administrative databases provide summative information that allows inter-institutional comparisons, they do not enable a detailed analysis of the case-mix factors at the individual level. This degree of data granularity is needed to unravel the underlying aetiologies of perioperative mortality, particularly preventable mortality. To date, no Australian study has analysed these factors at the national level.

The Australian and New Zealand Audit of Surgical Mortality (ANZASM) collects data on all in-hospital surgical mortality across Australia. It is managed by the Royal Australasian College of Surgeons (RACS) and includes an independent peer review process.^12^ This dataset represents a unique opportunity to better understand the factors associated with preventable mortality following oesophago-gastric cancer resections, with a view to inform system processes, and improve patient care in the future.

Accordingly, utilizing the ANZASM database, our objectives were: firstly to determine the causes of mortality following oesophago-gastric cancer resections in Australia; secondly, to quantify the proportion of potentially preventable deaths; and thirdly, to describe the underlying factors and clinical management issues that contributed to preventable mortality.

## Methods

### Study Design

A retrospective analysis of prospectively collected data from the ANZASM database was conducted between 1 January 2010 and 31 December 2020. This included all mortalities following elective pharyngo-laryngo-oesophagectomies, 3-stage oesophagectomies, 2-stage oesophagectomies, and total and subtotal gastrectomies for oesophago-gastric malignancies. Cases were excluded if they were under 18 years of age, had an emergency procedure, lacked assessor commentary, or had > 95% of missing data. The study was approved by the RACS ANZASM Committee.

### The ANZASM Process

A detailed description of the ANZASM has been reported elsewhere.^12^ Briefly, the ANZASM is an RACS directed national independent peer-reviewed audit of all in-patient surgical mortality in Australia. This initiative commenced in Western Australia in 2001. By 2010 it became nationwide, with 100% of public hospitals and the majority of private hospitals participating. This program was intended for quality-improvement and did not assign blame or establish negligence.

A standardized form was completed by the treating surgeon.^13^ Data were collected in a de-identified manner to ensure anonymity for both surgeons and patients. The collected data included patients’ demographics, insurance status, treatment location, comorbidities, perioperative risks assessment, and details of preoperative, intraoperative and postoperative management. Finally, the cause of death was assigned by the surgeon based on the clinical course and investigations. A coroner’s evaluation supported by a post-mortem examination could be undertaken when the cause of death was uncertain.

An independent peer review was then performed by a surgeon of the same specialty but from a different hospital. In this first-line assessment, the reviewing surgeon identified clinical management issues which they categorized by ascending levels of severity (‘Consideration’, ‘Concern’ or ‘Adverse event’*)*. Occasionally, a full case note review by another surgeon was requested by the first assessor to gain further clarity regarding the case. This second-line assessment also sought to identify and classify clinical management issues in the same manner. Both the first- and second-line assessors were asked to grade each clinical management issue with regards to its preventability (‘Definitely’, ‘Probably’, ‘Probably not’ or ‘Definitely not’ preventable) and potential contribution to mortality (‘Causative’, ‘May have caused’ or ‘No difference’). All assessment outcomes were then disclosed to the treating surgeon for feedback and professional development.

### Study Endpoints and Definitions

In this study, all postoperative complications were defined according to the Esophagectomy Complications Consensus Guidelines.^14^ To minimize bias, we analysed all clinical management issues regardless of severity. We defined potentially preventable mortality as any case with clinical management issues that were deemed ‘definitely’ or ‘probably’ preventable, and ‘causative’ or ‘may have caused’ the death of the patient. Non-preventable mortality was defined as a death: (1) without any clinical management issues, or (2) where these issues were deemed ‘probably not’ or ‘definitely not’ preventable, or (3) made ‘no difference’ to the likelihood of mortality. Clinical management issues from both first- and second-line assessors were combined. If both assessors described the same issue, this was counted once. Where a conflict arose between the assessors’ grading, the higher severity category was chosen.

### Thematic Analysis

Clinical management issues were classified into preoperative, intraoperative or postoperative categories. They were further classified into themes using a thematic analysis with a data-driven approach as described by Braun and Clarke.^15^ Two researchers (AF and PE) independently classified each assessor’s clinical management issues into the relevant themes. Differences were resolved through discussion with a senior author (DL).

### Statistical Analysis

For comparative analyses, categorical and continuous variables were analysed using Fisher’s exact test and Student’s *t*-tests, respectively. A two-tailed *p *< 0.05 and 95% confidence interval (CI) around the odds ratio (OR) that did not cross 1 was considered statistically significant. Statistical analyses were performed using Prism v9 (GraphPad Software, San Diego, CA, USA).

## Results

### Causes of Mortality

**Fig. 1:**
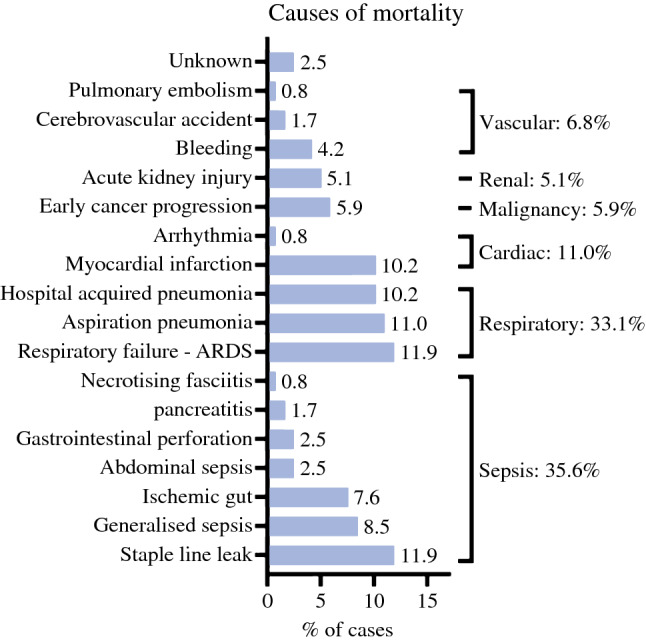
Causes of postoperative death following oesophago-gastric resection (*ARDS*: acute respiratory distress syndrome)

On reviewing the ANZASM database over a 10-year period, we identified 105 mortalities following major cancer resection of the upper gastrointestinal tract. These included 8 (7.6%) pharyngo-laryngo-oesophagectomies, 37 (35.2%) oesophagectomies, 30 (28.6%) total gastrectomies, and 30 (28.6%) subtotal gastrectomies. Baseline characteristics are presented in Table S1. The most common cause of death was cardio-respiratory in nature (44.1%), followed by sepsis (35.6%) secondary to anastomotic dehiscence, gut ischemia, bowel perforation, pancreatitis and necrotizing fasciitis (Fig. 1).

### Complications Preceding Mortality

Using the Esophagectomy Complications Consensus Guidelines,^14^ we analysed the incidence of different postoperative complications preceding each mortality (Table 1). Overall, 636 complications occurred in 105 patients. The median (IQR) number of complications per patient was 6 (4–8). The most common complications involved the cardio-respiratory system, followed by the gastrointestinal, urological, neurological, haematological and the integumentary systems. Generalized sepsis (45.7%), pneumonia (39.0%), staple line dehiscence (38.1%), acute kidney injury (28.6%), acute aspiration (21.9%) and bleeding (18.1%) were the most frequently reported complications (Table 1).

**Table 1:** Surgical complications prior to mortality

System	Complication description	N	% of 636 complications	% of 105 patients
Gastrointestinal	Leak from anastomosis, staple line, or localized conduit necrosis	40	6.3%	38.1%
	Bowel ischemia	18	2.8%	17.1%
	Feeding jejunostomy tube complication	11	1.7%	10.5%
	Liver dysfunction	11	1.7%	10.5%
	Anastomotic leak-small bowel	11	1.7%	10.5%
	Small-bowel obstruction	9	1.4%	8.6%
	Delayed conduit emptying requiring intervention	9	1.4%	8.6%
	Ileus (small-bowel dysfunction preventing or delaying enteral feeding)	7	1.1%	6.7%
	Anastomotic leak–pancreaticobiliary	5	0.8%	4.8%
	Pancreatitis	4	0.6%	3.8%
	Anastomotic leak-colorectal	3	0.5%	2.9%
	Pyloromyotomy/pyloroplasty complication	1	0.2%	1.0%
	*Clostridium difficile* infection	0	0.0%	0.0%
Infection	Other infections requiring antibiotics	54	8.5%	51.4%
	General sepsis	48	7.5%	45.7%
	Intrathoracic/intra-abdominal abscess	6	0.9%	5.7%
	Wound infection requiring opening wound or antibiotics	4	0.6%	3.8%
	Central intravenous line infection requiring removal or antibiotics	2	0.3%	1.9%
Neurological	Acute delirium	9	1.4%	8.6%
	Other neurological injury	5	0.8%	4.8%
	Recurrent laryngeal nerve injury	2	0.3%	1.9%
	Delirium tremens	0	0.0%	0.0%
Pulmonary	Pneumonia	41	6.4%	39.0%
	Respiratory failure	40	6.3%	38.1%
	Acute aspiration	23	3.6%	21.9%
	Pleural effusion requiring additional drainage procedure	14	2.2%	13.3%
	Acute respiratory distress syndrome	11	1.7%	10.5%
	Pneumothorax requiring intervention	4	0.6%	3.8%
	Tracheobronchial injury	4	0.6%	3.8%
	Atelectasis mucous plugging requiring bronchoscopy	2	0.3%	1.9%
	Air leak requiring drainage	2	0.3%	1.9%
Cardiac	Cardiac arrest requiring cardiopulmonary resuscitation	21	3.3%	20.0%
	Dysrhythmia-atrial	16	2.5%	15.2%
	Myocardial infarction	15	2.4%	14.3%
	Congestive heart failure requiring intervention	5	0.8%	4.8%
	Dysrhythmia-ventricular	1	0.2%	1.0%
	Pericarditis requiring intervention	0	0.0%	0.0%
Thromboembolic	Pulmonary embolism	4	0.6%	3.8%
	Stroke	4	0.6%	3.8%
	Deep vein thrombosis	3	0.5%	2.9%
	Peripheral thrombophlebitis	2	0.3%	1.9%
Urologic	Acute renal failure	30	4.7%	28.6%
	Acute renal failure requiring dialysis	19	3.0%	18.1%
	Urinary tract infection	2	0.3%	1.9%
	Urinary retention requiring reinsertion of urinary catheter	0	0.0%	0.0%
Wound/diaphragm	Acute abdominal wall dehiscence/hernia	4	0.6%	3.8%
	Thoracic wound dehiscence	2	0.3%	1.9%
	Acute diaphragmatic hernia	0	0.0%	0.0%
Other	Re-operation for reasons other than bleeding	46	7.2%	43.8%
	Multiple organ dysfunction syndrome	34	5.3%	32.4%
	Bleeding (intraluminal, intraabdominal, intrathoracic)	19	3.0%	18.1%
	Re-operation for bleeding	8	1.3%	7.6%
	Chyle leak	1	0.2%	1.0%

### Clinical Management Issues and Potentially Preventable Mortalities

**Fig. 2:**
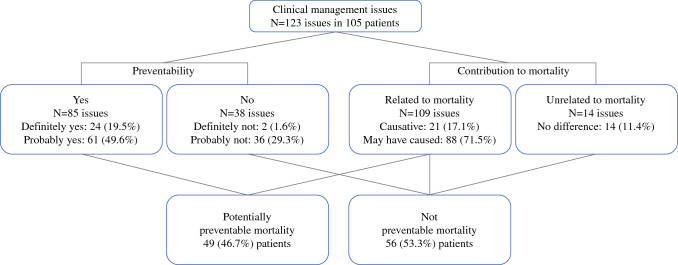
Breakdown of clinical management issues by preventability and contribution to mortality

In total, 123 clinical management issues were identified by first- or second-line assessors for each case. Second-line reviews were requested for 58 patients. Of the 105 mortalities, 66 (62.9%) patients had at least one clinical management issue flagged by assessors as an area of consideration (73, 59.3%), concern (33, 26.8%) or an overt adverse event (17, 13.8%). Overall, the median (IQR) number of clinical management issues per patient was 1 (0–2). Of the 123 clinical management issues, 109 (88.6%) were deemed to have directly caused or may have contributed to the death of the patient. Moreover, 69.1% of all clinical management issues were potentially preventable. Taken together, 49 (46.7%) mortalities in this cohort were potentially preventable (Fig. 2).

### Sensitivity Analysis of Mortality and Morbidity Excluding Pharyngo-Laryngo-Oesophagectomy Cases

Given that pharyngo-laryngo-oesophagectomy cases typically involve a different disease and treatment process than oesophago-gastric cancer cases, we performed a sensitivity analysis of mortality and morbidity excluding pharyngo-laryngo-oesophagectomy from the overall cohort. Of the remaining 97 cases, the most common cause of death (Fig. S1) was still cardio-respiratory in nature (43.1%), followed by sepsis (36.8%). These 97 patients also had a comparable complication profile to the parental cohort (Table S2). Moreover, of the 8 mortalities following pharyngo-laryngo-oesophagectomy, 4 (50.0%) were deemed potentially preventable. This is in keeping with the overall rate of potentially preventable mortality. Given that excluding pharyngo-laryngo-oesophagectomy cases did not significantly affect the profile of morbidity, mortality and, particularly, potentially preventable mortality, we decided to include pharyngo-laryngo-oesophagectomy cases into all subsequent mechanistic analyses of potentially preventable mortalities.

### Factors Associated with Potentially Preventable Mortalities

To better understand the factors associated with potentially preventable mortality following oesophago-gastric resections, we compared the characteristics of 49 patients whose deaths were potentially preventable with those of the 56 patients whose deaths were deemed not preventable (Table 2). These two groups were similar with respect to their baseline characteristics, co-morbidities, perioperative mortality risk and surgical approach. However, patients with potentially preventable mortality had a significantly higher number of complications (Tables 2 and S3). These included generalized sepsis (59.2% vs 33.9%, *p *= 0.011), multiorgan dysfunction syndrome (40.8% vs 25.0%, *p* = 0.042), re-operation (63.3% vs 41.1%, *p *= 0.031), small-bowel obstruction (14.3% vs 3.6%, *p* = 0.025), delayed conduit emptying that required reintervention (14.3% vs 3.6%, *p *= 0.025) and jejunostomy-related issues (18.4% vs 3.6%, *p *= 0.022). Importantly, this group also had a significantly higher number of clinical management issues per patient [median (IQR): 2 (1–3) vs 0 (0–1), *p *< 0.001), which adversely impacted on preoperative (30.6% vs 7.1%, *p *= 0.002), intraoperative (18.4% vs 5.4%, *p* = 0.037), and postoperative (51.0% vs 17.9%, *p *< 0.001) patient care (Table 2).

**Table 2:** Factors associated with preventable and not preventable mortality

Characteristics	Potential preventable mortality *N* = 49	Not preventable mortality *N* = 56	*p* value
Age, years, mean (SD)	69.9 (11.1)	73.7 (9.9)	0.056
Female, *n* (%)	17 (34.7)	9 (16.1)	0.041
Time to death, days, median (IQR)	20 (10–41)	16 (8–30)	0.336
Year of death, ≤ 2015, *n* (%)	25 (51.0)	38 (67.9)	0.110
Regionality, *n* (%)			1.000
Regional centres	19 (38.8)	21 (37.5)	
Capital city centres	30 (61.2)	35 (62.5)	
Hospital status, *n* (%)			1.000
Private	16 (32.7)	19 (33.9)	
Public	33 (67.3)	37 (66.1)	
Patient insurance status, *n* (%)			1.000
Private	19 (38.8)	21 (37.5)	
Public	30 (61.2)	35 (62.5)	
Body mass index, > 30 kg/m^2^, *n* (%)	8 (16.3)	11 (19.6)	0.801
Smoker, *n* (%)	3 (6.1)	1 (1.8)	0.337
ASA, median (IQR)	3 (2–3)	3 (2–3)	0.184
Preoperative risk of death, graded by surgeon, *n* (%)			0.097
Minimal	5 (10.2)	1 (1.8)	
Small	20 (40.8)	18 (32.1)	
Moderate	21 (42.9)	28 (50.0)	
Considerable	3 (6.1)	9 (16.1)	
Operative details			
Consultant operating, yes, *n* (%)	41 (83.7)	48 (85.7)	0.792
Consultant assisting, yes, *n* (%)	10 (20.4)	18 (32.1)	0.192
Consultant in theatre, yes, *n* (%)	43 (87.8)	53 (94.6)	0.299
Length of surgery, h, mean (SD)	4.6 (2.3)	4.2 (2.1)	0.389
Operation, *n* (%)			0.668
Pharyngo-laryngo-oesophagectomy	4 (8.2)	4 (7.1)	
Oesophagectomy, 3 stage	11 (22.4)	8 (14.3)	
Oesophagectomy, 2 stage	8 (16.3)	10 (17.9)	
Total gastrectomy	11 (22.4)	19 (33.9)	
Subtotal gastrectomy	15 (30.6)	15 (26.8)	
Postoperative complications, number/patient, median (IQR)			
Total	7 (5–9)	5 (3–7)	0.009
Re-operations	1 (0–1)	0 (0–1)	0.033
Multiple organ dysfunction syndrome	1 (0–1)	0 (0–1)	0.043
Gastrointestinal	1 (1–2)	0 (0–1)	< 0.001
Infection	1 (1–2)	1 (0–2)	0.004
Neurological	0 (0–0)	0 (0–0)	0.792
Pulmonary	1 (0–3)	1 (0–2)	0.326
Cardiac	0 (0–1)	0 (0–1)	0.466
Thromboembolic	0 (0–0)	0 (0–0)	0.321
Urological	0 (0–1)	0 (0–1)	0.614
Wound/diaphragm	0 (0–0)	0 (0–0)	0.095
Other	1 (1–2)	1 (0–2)	0.109
Clinical management issues, number/patient, median (IQR)	2 (1–3)	0 (0–1)	< 0.001
Patients with clinical management issues, n (%)			
Preoperative	15 (30.6)	4 (7.1)	0.002
Intraoperative	9 (18.4)	3 (5.4)	0.037
Postoperative	25 (51.0)	10 (17.9)	< 0.001

*ASA* American Society of Anesthesiology, *IQR* interquartile range, *SD* standard deviation

### Thematic Analysis of Potentially Preventable Clinical Management Issues

To gain further insights into the aetiology of potentially preventable mortalities following oesophago-gastric resections, we performed a thematic analysis of all 103 clinical management issues that occurred in patients whose deaths were potentially preventable. Overall, 38 (36.9%), 12 (11.7%) and 53 (51.4%) of these issues were categorized into preoperative, intraoperative and postoperative themes, respectively.

Table 3 details the clinical management issues which impacted preoperative care. Of these, inadequate surgical assessment, planning, and/or optimization of patient fitness for surgery (9 cases, 23.7%), inappropriate decision to offer surgery (11 cases, 28.9%) and incorrect choice or approach to an operation (11 cases, 28.9%), were the most commonly identified and potentially preventable deficiencies in the preoperative period.

**Table 3:** Preventable preoperative clinical management issues

Themes	*n* (%)	Assessor’s comments
Patient workup	9 (23.7)	Inadequate surgical planning, assessment and/or optimization of patient fitness for surgery
	1 (2.6)	Timing of preoperative staging scans resulted in missed rapid progression of oesophageal cancer
	1 (2.6)	Carcinoid crisis management not considered or discussed with specialist preoperatively
Treatment delay	3 (10.7)	Delay to operation
Seniority of staff and availability of resources	1 (2.6)	Failure to transfer/escalate care-Decision to operate rather than transfer to larger centre
	1 (2.6)	Failure to transfer/escalate care-Inadequate surgical technical ability and resource availability at centre
Treatment decision making	1 (2.6)	Inappropriately high dose of radiotherapy
	11 (28.9)	Inappropriate choice or approach of operation
	11 (28.9)	Inappropriate decision to offer or perform surgery

Table 4 details the clinical management issues which impacted intraoperative care. Of these, multiple unique events within the themes of technical error (8 cases, 66.7%), incorrect decision-making (3 cases, 25.0%) and lack of senior surgeon availability (1 case, 8.3%) were identified as potentially preventable contributors to mortality.

**Table 4:** Preventable intraoperative clinical management issues

Themes	*n* (%)	Assessor’s comments
Technical error	3 (25.0)	Chyle leak due to injury or failure to ligate thoracic duct
	1 (8.3)	Error in dissection plane causing injury to superior mesenteric vein branches with bleeding and inadequate haemostasis
	1 (8.3)	Iatrogenic duodenal injury
	1 (8.3)	Intraoperative bleeding due to iatrogenic injury to portal vein
	1 (8.3)	Intraoperative bleeding due to iatrogenic injury to left-sided SVC
	1 (8.3)	Confusion during reconstruction with malalignment of conduit
Seniority of staff and availability of resources	1 (8.3)	Inappropriate absence of consultant at re-operation with two critical events
Treatment decision making	1 (8.3)	No rapid sequence induction despite pre-induction vomit
	1 (8.3)	Decision to proceed with major surgery after anaphylaxis soon after induction
	1 (8.3)	Decision to re-anastomose rather than oesophagostomy in return to theatre for septic patient

Table 5 details the clinical management issues which impacted postoperative care. Of these, failure to recognize a deteriorating patient and diagnose the underlying cause (23 cases, 43.4%), inappropriate treatment decision-making (22 cases, 41.5%) and delays in the delivery of critical services (8 cases, 15.1%) were the most common themes identified in the postoperative period that were potentially preventable.

**Table 5:** Preventable postoperative clinical management issues

Themes	*n* (%)	Assessor’s comments
Failure to recognize and respond to deterioration	11 (20.8)	Delay in diagnosis of leak
	3 (5.7)	Delay in diagnosis of respiratory failure
	2 (3.8)	Delay in diagnosis of conduit necrosis
	2 (3.8)	Delay in diagnosis of tamponade
	2 (3.8)	Delay in diagnosis of a deteriorating patient
	1 (1.9)	Delay in diagnosis of anastomotic stenosis
	1 (1.9)	Delay in diagnosis and management of ileus
	1 (1.9)	Delay in diagnosis of bowel ischemia and sepsis
Treatment decision making	4 (7.5)	Multiple postoperative care deficiencies
	3 (5.7)	Failure to secure airway leading to aspiration during insertion of nasojejunal feeding tube
	2 (3.8)	Inappropriate management of pneumonia
	1 (1.9)	Inappropriate use of feeding jejunostomy
	1 (1.9)	Early feeding leading to aspiration
	1 (1.9)	Inappropriate intensive care unit management of oliguria and respiration
	1 (1.9)	Inappropriate management of fluid overload
	1 (1.9)	Inappropriate management of pain
	1 (1.9)	Inappropriate management of nutrition
	1 (1.9)	Inappropriate management of epidural related hypotension
	1 (1.9)	Post-operative hypotension managed by inotropes rather than fluids inappropriately
	1 (1.9)	Premature discharge from ICU of high-risk patient
	1 (1.9)	Use of CPAP post-oesophagectomy
	1 (1.9)	Decision to stent oesophago-tracheal fistula rather than thoracotomy and re-operation
	1 (1.9)	Failure to cease feeds after vomiting
	1 (1.9)	Patient not managed in intensive care unit postoperatively
Treatment delay	4 (7.5)	Delay in accessing return to emergency theatre
	1 (1.9)	Delay in management of chyle leak
	1 (1.9)	Delay to required bronchoscopy
	1 (1.9)	Delay in re-admission to intensive care
	1 (1.9)	Delay in chest physiotherapy
Communication issue	1 (1.9)	Delay in informing surgeon of deterioration
	1 (1.9)	Difficult resuscitation with efforts ceased prior to surgeon review
	1 (1.9)	Poor communication between surgical team and the intensive care unit regarding hypotensive patient
Treatment error	1 (1.9)	Endotracheal tube errantly removed in intensive care unit which likely contributed to hypoxic arrest
	1 (1.9)	Haemorrhage due to erosion of drain into intercostal vessel

## Discussion

Using a national audit database, we identified with high granularity the underlying causes, complications and management issues that contributed to mortality following oesophago-gastric cancer surgery in Australia. Our key findings were: (1) approximately 50% of deaths were potentially preventable; (2) of these, most mortalities were preceded by multiple complications and clinical management issues; and (3) potentially preventable mortalities were associated with significantly higher rates of complications and clinical management issues than non-preventable mortalities. Moreover, our thematic analysis highlighted recurrent areas of deficiency to better direct future quality-improvement efforts.

Based on a national postoperative mortality rate of 3.5% for oesophago-gastric cancer resections in Australia,^2^ the 105 mortalities presented here were derived from approximately 3000 surgeries performed in multiple centres over 10 years. While each death is likely to be well considered within its respective units, a collective review of all these cases have not been available until now. Thus, our study is unique in its dissection of the clinical events leading to potentially preventable mortality after oesophago-gastric cancer surgery.

Our analysis showed that potentially preventable mortality was characterized by an increasing number of complications per patient (median, 7 per patient), higher rates of re-operation, sepsis and multiorgan failure, as well as significantly more clinical management issues at every phase of patient care (Tables 2 and S2). Importantly, it is difficult to predict potentially preventable mortality. Indeed, we noted a trend towards a lower preoperative risk of death in this group compared with the non-preventable mortality group. These findings evoke Reason’s Swiss Cheese Model for patient safety, which proposed that harm results from the alignment of multiple inherent weaknesses within a continuum of care.^16^ Synonymously, in most cases within our cohort, there were multiple opportunities for intervention that may have averted complications and death. Therefore, while it is generally accepted that oesophago-gastric cancer surgery carries a significant morbidity risk,^17^ the key is to implement processes to safeguard against omissions and correct commissions, however small, to avoid the conversion of morbidity into mortality.

Broadly, the themes identified from our analysis of clinical management issues overlapped with other examinations of mortality following cholecystectomy,^18^ neurosurgery,^19^ cardiothoracic surgery,^20^ pancreaticoduodenectomy^21^ and hepatectomy.^22^

The main themes identified in preoperative care were insufficient patient workup and poor decision-making. Within these themes, we found that suboptimal assessment of patient fitness, inappropriate decisions to offer surgery, incorrect procedural approaches and inadequate personnel or facility support were recurrent issues. These themes highlight the importance of patient selection in oesophago-gastric cancer surgery. Case selection extends beyond tumour staging and involves understanding each patient’s perioperative risks as well as their physiological reserve to surmount any complications that arise. This is critically important as most oesophago-gastric cancer patients have poor baseline fitness^23^ and are frequently malnourished at diagnosis.^24^ Moreover, their body composition and functional status are further impaired by neoadjuvant therapies,^25^ putting them at increased risk of morbidity and mortality. To enable adequate case selection, there are now validated risk prediction models and multi-faceted prehabilitation programs tailored for this patient population.^26^^,^^27^ In particular, a prehabilitation program should objectively assess (at baseline), optimize, and reassess (post-optimization) each patient’s medical, physical, nutritional and psychological fitness for surgery.^27^ Although various facets of prehabilitation are currently under investigation, evidence suggests that such programs improve outcomes for surgical patients with oesophago-gastric cancer.^27^ Notably, the benefit of prehabilitation is most pronounced in reducing cardio-respiratory complications,^27^ which were responsible for most of the morbidity and mortality in our cohort. Ideally, outcomes from risk prediction and prehabilitation should be incorporated into cancer-board discussions to guide patient management. In this way, the intent of treatment, as well as the approach, timing and location of surgery, takes into consideration not only tumour biology, but also patient physiology.

The main themes identified in intraoperative care were the absence of a senior surgeon (especially at re-operation), incorrect decision making, and technical errors. While intraoperative clinical management issues contributed the least to preventable mortality, they emphasized the relative complexities of an oesophago-gastric cancer resection, particularly if undertaken in a high-stress environment, where the surgeon is at risk of tunnel vision and cognitive overload.^28^ In this context, mistakes in decision-making and technical errors can have significant repercussions for patient outcomes. To address these issues, it is now recognized that surgical safety checklists,^29^ availability of a highly functioning team^30^ and close consultant supervision improve outcomes for complex surgeries.^31^^,^^32^ Practice guidelines from the Royal College of Surgeons of England describes the importance of an ‘expert team’ rather than an ‘expert surgeon’ in minimizing intraoperative errors.^33^ A highly functioning team consists of personnel who are confident in their own abilities and are familiar with the operation, other team members, and theatre resources. Accordingly, this team is able to anticipate and compensate for mishaps that may occur in theatre, thereby decreasing the rate and impact of errors.^33^ Markar et al. showed that surgeon experience is directly associated with patient mortality following oesophago-gastric cancer resections.^34^ They and others propose that complex surgeries should be undertaken by two surgeons, either in a partnership or mentor-mentee capacity, to facilitate shared decision-making, reduce cognitive overload, and manage errors when they occur.^34–36^

The main themes identified in postoperative care were failure to recognize the deteriorating patient, incorrect decision making and treatment delays. Consistent with other studies, postoperative clinical management issues contributed the most to preventable mortality.^19–22^ While close consultant input and increased vigilance by all team members for signs of deterioration are undoubtedly important in the postoperative period, it is recognized that senior clinicians may not always be on site, and junior team members may be inexperienced in recognizing (or acting on) these signs. To address these issues, many centres have implemented enhanced recovery pathways.^37^ Although these pathways vary among institutions, studies have demonstrated their efficacy in decreasing postoperative complications.^37^^,^^38^ Moreover, these pathways serve as a template for uneventful recovery following oesophago-gastric cancer surgery. Any deviation from the expected clinical course may facilitate early diagnosis and management of potential complications. Additionally, as these programs are typically multidisciplinary and well-documented within an institution, they can improve shared decision-making and minimize misunderstanding between teams. In the authors’ experience, embedding a dedicated cancer care coordinator or nurse practitioner within these programs further enhances communication between treatment teams and improves the quality of care.

It has been argued that the centralization of cancer services can reduce in-hospital mortality. Indeed, recent analyses of administrative datasets by our group have demonstrated a hospital volume-outcome relationship for Australia, in favour of high-volume centres (≥ 12 resections per year per centre) producing the lowest in-hospital mortality (1.6%).^7^ This is consistent with experiences from Europe, Asia and the USA.^4–6^ It is suggested that improved performance in higher volume centres can be partly attributable to staff members being more familiar with managing surgical patients with oesophago-gastric cancer. These centres may have system processes in place to better select and optimize patients, as well as to recognize problems and rescue early. Moreover, system improvements within a hospital may be easier to implement when there is a higher patient throughput. Despite these potential benefits, efforts towards the centralization of cancer services within Australia have faced numerous challenges. These include resistive societal attitudes, mixed public/private health services, state-governed healthcare, and vast geographical distances with a sparse population density. Additionally, issues surrounding the definition of high-volume, and the relative importance of surgeon versus hospital volume needs to be resolved. Fortunately, in-hospital mortality following oesophago-gastric cancer resections has steadily declined over the last 30 years across Australia. This suggests that, even in the absence of centralization, local quality-improvement efforts across the preoperative, intraoperative and postoperative domains are critically important to minimize surgical mortality.

This study has several limitations. First, the assessors’ comments were subjective. However, we found that for most potentially preventable mortalities, there were two independent assessors. Second, the ANZASM database is limited to patients who died. It is not a national registry for all patients who undergo oesophago-gastric cancer surgery. Therefore, we could not provide a population estimate for some of the comparisons. Third, participation from the private sector is incomplete. We recognize that the models of care and patient risk profiles are different between public and private sectors. Fourth, the overall sample size is low despite near-complete national participation in the ANZASM process. Fifth, this study drew on cases across a 10-year period. It is possible that clinical practices may have changed over time in these centres. Finally, the scope of the data obtained by ANZASM does not capture all case note details, such as surgical approach; however, this information was available to all second-line assessors.

Overall, the findings from this study can inform surgical practice and training, and can be used as a basis for prioritizing quality improvement initiatives. Moreover, many of our findings can be applied to other surgical specialties,^18–22^ and other disciplines may also benefit from a similar analysis of mortality data.

## Supplementary Information

Below is the link to the electronic supplementary material.

Supplementary file1 (DOCX 55 KB)
