# Supplementary material for: Understanding Potentially Preventable Mortality Following Oesophago-Gastric Cancer Surgery: Analysis of a National Audit of Surgical Mortality
Source: Ann Surg Oncol. 2023 May 8;30(8):4950–61. doi: 10.1245/s10434-023-13571-8 (PMC10319683; doi:10.1245/s10434-023-13571-8)
Supplement: Supplementary file 1 — Supplementary file1 (DOCX 55 KB) [file 10434_2023_13571_MOESM1_ESM.docx]

**Supplementary material**

**Table S1.** Baseline characteristics of all mortalities

| **Characteristics** | **All mortalities**  **N=105** |
| --- | --- |
| Age, years, mean (SD) | 71.7 (10.5) |
| Female, n (%) | 26 (24.8) |
| Time to death, days, median (IQR) | 17 (9-35) |
| Year of death, ≤2015, n (%) | 63 (60.0) |
| Regionality, n (%) |  |
| Regional centers | 40 (38.1) |
| Capital city centers | 65 (61.9) |
| Hospital status, n (%) |  |
| Private | 35 (33.3) |
| Public | 70 (66.7) |
| Patient insurance status, n (%) |  |
| Private | 40 (38.1) |
| Public | 65 (61.9) |
| Body mass index, >30 kg/m^2^, n (%) | 19 (18.1) |
| Smoker, n (%) | 4 (3.8) |
| ASA, median (IQR) | 3 (2-3) |
| Preoperative risk of death, graded by surgeon, n (%) |  |
| Minimal | 6 (5.7) |
| Small | 38 (36.2) |
| Moderate | 49 (46.7) |
| Considerable | 12 (11.4) |
| Co-morbidities, n (%) |  |
| Cardiovascular diseases | 47 (44.8) |
| Chronic respiratory diseases | 41(39.0) |
| Chronic renal injury | 18 (17.1) |
| Chronic hepatic disease | 5 (4.8) |
| Neurological diseases | 11 (10.5) |
| Advanced malignancy | 28 (26.7) |
| Diabetes | 14 (13.3) |
| Operative details |  |
| Consultant operating, yes, n (%) | 89 (84.8) |
| Consultant assisting, yes, n (%) | 28(26.7) |
| Consultant in theatre, yes, n (%) | 96 (91.4) |
| Length of surgery, hr, mean (SD) | 4.4 (2.2) |
| Operation, n (%) |  |
| Pharyngo-laryngo-esophagectomy | 8 (7.6) |
| Esophagectomy, 3 stage | 19 (18.1) |
| Esophagectomy, 2 stage | 18 (17.1) |
| Total gastrectomy | 30 (28.6) |
| Subtotal gastrectomy | 30 (28.6) |

ASA: American Society of Anesthesiology, IQR: interquartile range, SD: standard deviation

**Figure S1.** Causes of postoperative death following esophago-gastric resection excluding patient who undergone pharyngo-laryngo-esophagectomy (ARDS: acute respiratory distress syndrome)

**Table S2.** Surgical complications prior to mortality

| **System** | **Complication description** | **N** | **% Of 580** | **% Of 97** |
| --- | --- | --- | --- | --- |
|  |  |  | **complications** | **patients** |
| Gastrointestinal | Leak from anastomosis, staple line, or localized conduit necrosis | 39 | 6.70% | 40.20% |
|  | Bowel ischemia | 16 | 2.80% | 16.50% |
|  | Feeding Jejunostomy tube complication | 9 | 1.60% | 9.30% |
|  | Liver dysfunction | 10 | 1.70% | 10.30% |
|  | Anastomotic leak - small bowel | 10 | 1.70% | 10.30% |
|  | Small bowel obstruction | 8 | 1.40% | 8.20% |
|  | Delayed conduit emptying requiring intervention | 8 | 1.40% | 8.20% |
|  | Ileus (small bowel dysfunction preventing or delaying enteral feeding) | 6 | 1.00% | 6.20% |
|  | Anastomotic leak – pancreaticobiliary | 3 | 0.50% | 3.10% |
|  | Pancreatitis | 4 | 0.70% | 4.10% |
|  | Anastomotic leak - colorectal | 1 | 0.20% | 1.00% |
|  | Pyloromyotomy/pyloroplasty complication | 1 | 0.20% | 1.00% |
|  | Clostridium difficile infection | 0 | 0.00% | 0.00% |
| Infection | Other infections requiring antibiotics | 49 | 8.40% | 50.50% |
|  | General sepsis | 46 | 7.90% | 47.40% |
|  | Intrathoracic/Intra-abdominal abscess | 6 | 1.00% | 6.20% |
|  | Wound infection requiring opening wound or antibiotics | 4 | 0.70% | 4.10% |
|  | Central intravenous line infection requiring removal or antibiotics | 2 | 0.30% | 2.10% |
| Neurological | Acute delirium | 8 | 1.40% | 8.20% |
|  | Other neurological injury | 4 | 0.70% | 4.10% |
|  | Recurrent laryngeal nerve injury | 1 | 0.20% | 1.00% |
|  | Delirium tremens | 0 | 0.00% | 0.00% |
| Pulmonary | Pneumonia | 37 | 6.40% | 38.10% |
|  | Respiratory failure | 36 | 6.20% | 37.10% |
|  | Acute aspiration | 20 | 3.40% | 20.60% |
|  | Pleural effusion requiring additional drainage procedure | 12 | 2.10% | 12.40% |
|  | Acute respiratory distress syndrome | 10 | 1.70% | 10.30% |
|  | Pneumothorax requiring intervention | 4 | 0.70% | 4.10% |
|  | Tracheobronchial injury | 4 | 0.70% | 4.10% |
|  | Atelectasis mucous plugging requiring bronchoscopy | 1 | 0.20% | 1.00% |
|  | Air leak requiring drainage | 2 | 0.30% | 2.10% |
| Cardiac | Cardiac arrest requiring cardiopulmonary resuscitation | 19 | 3.30% | 19.60% |
|  | Dysrhythmia - atrial | 14 | 2.40% | 14.40% |
|  | Myocardial infarction | 14 | 2.40% | 14.40% |
|  | Congestive heart failure requiring intervention | 4 | 0.70% | 4.10% |
|  | Dysrhythmia - ventricular | 1 | 0.20% | 1.00% |
|  | Pericarditis requiring intervention | 0 | 0.00% | 0.00% |
| Thromboembolic | Pulmonary embolism | 4 | 0.70% | 4.10% |
|  | Stroke | 4 | 0.70% | 4.10% |
|  | Deep vein thrombosis | 3 | 0.50% | 3.10% |
|  | Peripheral thrombophlebitis | 2 | 0.30% | 2.10% |
| Urologic | Acute renal failure | 28 | 4.80% | 28.90% |
|  | Acute renal failure requiring dialysis | 18 | 3.10% | 18.60% |
|  | Urinary tract infection | 2 | 0.30% | 2.10% |
|  | Urinary retention requiring reinsertion of urinary catheter | 0 | 0.00% | 0.00% |
| Wound/diaphragm | Acute abdominal wall dehiscence/hernia | 3 | 0.50% | 3.10% |
|  | Thoracic wound dehiscence | 2 | 0.30% | 2.10% |
|  | Acute diaphragmatic hernia | 0 | 0.00% | 0.00% |
| Other | Reoperation for reasons other than bleeding | 42 | 7.20% | 43.30% |
|  | Multiple organ dysfunction syndrome | 32 | 5.50% | 33.00% |
|  | Bleeding (intraluminal, intraabdominal, intrathoracic) | 18 | 3.10% | 18.60% |
|  | Reoperation for bleeding | 8 | 1.40% | 8.20% |
|  | Chyle leak | 1 | 0.20% | 1.00% |
|  | Chyle leak | 1 | 0.20% | 1.00% |

**Table S3.** Postoperative complications with preventable and not preventable mortality

| **System** | **Complication description** | **Potential preventable**  **mortality**  **N=49** | **Not preventable**  **mortality**  **N=56** | ***p***  **value** |
| --- | --- | --- | --- | --- |
| Gastrointestinal,  n (%) | Leak from anastomosis, staple line, or localized conduit necrosis | 21 (42.9) | 19 (33.9) | 0.422 |
|  | Bowel ischemia | 6 (12.2) | 12 (21.4) | 0.300 |
|  | Feeding Jejunostomy tube complication | 9 (18.4) | 2 (3.6) | **0.022** |
|  | Liver dysfunction | 8 (16.3) | 3 (5.4) | 0.108 |
|  | Anastomotic leak - small bowel | 4 (8.2) | 7 (12.5) | 0.537 |
|  | Small bowel obstruction | 7 (14.3) | 2 (3.6) | **0.025** |
|  | Delayed conduit emptying requiring intervention | 7 (14.3) | 2 (3.6) | **0.025** |
|  | Ileus (small bowel dysfunction preventing or delaying enteral feeding) | 5 (10.2) | 2 (3.6) | 0.247 |
|  | Pancreatitis | 2 (4.1) | 2 (3.6) | 1.000 |
|  | Pyloromyotomy/pyloroplasty complication | 1 (2.0) | 0 (0.0) | 1.000 |
|  | Clostridium difficile infection | 0 (0.0) | 0 (0.0) | 1.000 |
| Infection, n (%) | Other infections requiring antibiotics | 31 (63.3) | 23 (41.1) | **0.031** |
|  | General sepsis | 29 (59.2) | 19 (33.9) | **0.011** |
|  | Intrathoracic/Intra-abdominal abscess | 4 (8.2) | 2 (3.6) | 0.414 |
|  | Wound infection requiring opening wound or antibiotics | 3 (6.1) | 2 (3.6) | 0.662 |
|  | Central intravenous line infection requiring removal or antibiotics | 0 (0.0) | 2 (3.6) | 0.497 |
| Neurological, n (%) | Acute delirium | 4 (8.2) | 5 (8.9) | 1.000 |
|  | Other neurological injury | 3 (6.1) | 2 (3.6) | 0.662 |
|  | Recurrent laryngeal nerve injury | 1 (2.0) | 1 (1.8) | 1.000 |
|  | Delirium tremens | 0 (0.0) | 0 (0.0) | - |
| Pulmonary, n (%) | Pneumonia | 17 (34.7) | 24 (42.9) | 0.428 |
|  | Respiratory failure | 21 (42.9) | 19 (33.9) | 0.422 |
|  | Acute aspiration | 11 (22.4) | 12 (21.4) | 1.000 |
|  | Pleural effusion requiring additional drainage procedure | 9 (18.4) | 5 (8.9) | 0.249 |
|  | Acute respiratory distress syndrome | 6 (12.2) | 5 (8.9) | 0.752 |
|  | Pneumothorax requiring intervention | 3 (6.1) | 1 (1.8) | 0.337 |
|  | Tracheobronchial injury | 3 (6.1) | 1 (1.8) | 0.337 |
|  | Atelectasis mucous plugging requiring bronchoscopy | 1 (2.0) | 1 (1.8) | 1.000 |
|  | Air leak requiring drainage | 2 (4.1) | 0 (0.0) | 0.215 |
| Cardiac, n (%) | Cardiac arrest requiring cardiopulmonary resuscitation | 10 (20.4) | 11 (19.6) | 1.000 |
|  | Dysrhythmia - atrial | 9 (18.4) | 7 (12.5) | 0.429 |
|  | Myocardial infarction | 2 (4.1) | 13 (23.2) | **0.005** |
|  | Congestive heart failure requiring intervention | 1 (2.0) | 4 (7.1) | 0.369 |
|  | Dysrhythmia - ventricular | 1 (2.0) | 0 (0.0) | 0.467 |
|  | Pericarditis requiring intervention | 0 (0.0) | 0 (0.0) | - |
| Thromboembolic, n (%) | Pulmonary embolism | 2 (4.1) | 2 (3.6) | 1.000 |
|  | Stroke | 1 (2.0) | 3 (5.4) | 0.621 |
|  | Deep vein thrombosis | 1 (2.0) | 2 (3.6) | 1.000 |
|  | Peripheral thrombophlebitis | 0 (0.0) | 2 (3.6) | 0.497 |
| Urologic, n (%) | Acute renal failure | 13 (26.5) | 17 (30.4) | 0.829 |
|  | Acute renal failure requiring dialysis | 8 (16.3) | 11 (19.6) | 0.801 |
|  | Urinary tract infection | 0 (0.0) | 2 (3.6) | 0.497 |
|  | Urinary retention requiring reinsertion of urinary catheter | 0 (0.0) | 0 (0.0) | - |
| Wound/diaphragm, n (%) | Acute abdominal wall dehiscence/hernia | 3 (6.1) | 1 (1.8) | 0.337 |
|  | Thoracic wound dehiscence | 2 (4.1) | 0 (0.0) | 0.215 |
|  | Acute diaphragmatic hernia | 0 (0.0) | 0 (0.0) | - |
| Other, n (%) | Reoperation overall | 31 (63.3) | 23 (41.1) | **0.031** |
|  | Reoperation for reasons other than bleeding | 26 (53.1) | 20 (35.7) | **0.037** |
|  | Reoperation for bleeding | 5 (10.2) | 3 (5.4) | **0.469** |
|  | Multiple organ dysfunction syndrome | 20 (40.8) | 14 (25.0) | **0.042** |
|  | Bleeding (intraluminal, intraabdominal, intrathoracic) | 10 (20.4) | 9 (16.1) | 0.618 |
|  | Chyle leak | 4 (8.2) | 1 (1.8) | 0.182 |
